# Cutaneous Metastasis of Rectal Cancer as a Diagnostic Challenge: A Clinical Case and Literature Review

**DOI:** 10.3390/diagnostics14212420

**Published:** 2024-10-30

**Authors:** Ekaterina Zelenova, Tatiana Belysheva, Denis Sofronov, Vera Semenova, Galimat Radjabova, Yana Vishnevskaya, Irina Kletskaya, Elena Sharapova, Ivan Karasev, Denis Romanov, Malika Denieva, Nikolay Petrochenko, Timur Valiev, Tatiana Nasedkina

**Affiliations:** 1 N.N. Blokhin National Medical Research Center of Oncology, Ministry of Health of the Russian Federation, 115478 Moscow, Russia; zelenovayeye@gmail.com (E.Z.); klinderma@bk.ru (T.B.); mdsofronov@gmail.com (D.S.); sulpiridum@yandex.ru (V.S.); yana_vishn@list.ru (Y.V.); sharapovae.v@yandex.ru (E.S.); i.karasev@ronc.ru (I.K.); petrochenko_nikolayy@rambler.ru (N.P.); timurvaliev@mail.ru (T.V.); 2 Engelhardt Institute of Molecular Biology, Russian Academy of Sciences, 119991 Moscow, Russia; 3 I.M. Sechenov First Moscow State Medical University, Ministry of Health of the Russian Federation, 119048 Moscow, Russia; galimatradjabova2001@gmail.com; 4 Russian Children’s Clinical Hospital, Pirogov Russian National Research Medical University, Ministry of Health of the Russian Federation, 119571 Moscow, Russia; ikletskaya@gmail.com; 5 Center of Innovative Medical Technologies, 119991 Moscow, Russia; romanovronc@gmail.com; 6 Department of Polyclinic Therapy, Chechen State University, 364061 Grozny, Russia; denieva54@mail.ru

**Keywords:** rectal cancer, cutaneous metastases, immunohistochemistry, somatic mutations, next generation sequencing

## Abstract

**Background/Objectives:** Metastatic colorectal cancer remains a fatal disease, with a 5-year survival rate lower than 15%. The most common metastatic sites are the lungs and the liver, while skin metastases are very rare and often indicate a poor prognosis with a lower survival rate. Methods. Herein, we present the clinical case of a 62-year-old female patient with rectal cancer metastases to the skin of the anogenital and abdominal regions, diagnosed 2 years after completion of treatment of the underlying disease. **Results:** Histological examination of the skin lesions revealed adenocarcinoma, and expression of the same immunohistochemical markers was also found in the primary tumor and in the cutaneous metastases. However, next-generation sequencing demonstrated differences in the mutational profiles of the primary tumor and metastasis to the skin. Somatic mutations in the *APC*, *TP53*, and *PTPN11* genes were revealed in primary rectal adenocarcinoma, but another pathogenic *TP53* mutation and a frameshift variant in the *DYNC1I1* gene were found in cutaneous metastases. The patient underwent several courses of FOLFOX6 chemotherapy in combination with bevacizumab, but the treatment was unsuccessful. An analysis of 50 clinical cases from the literature concerning various manifestations of cutaneous metastases of rectal cancer showed a median survival of 8.5 months from the time of detection of the skin lesions. **Conclusions:** In this regard, careful skin examination of patients with rectal cancer and timely detection of cutaneous metastases are essential steps in the follow-up of patients who have undergone treatment of the primary tumor.

## 1. Introduction

According to the World Health Organization (WHO), in 2022, colorectal cancer (CRC) ranked third in incidence and second in mortality among all neoplasms in the world [1]. In Russia, in 2022, CRC (colon cancer and rectal cancer, including rectosigmoid junction and anus cancer) ranked third among all malignant neoplasms (75,791 cases). During the same time, 31,442 newly diagnosed cases of rectal cancer (RC) were registered, accounting for 5.0% of all malignant neoplasms and 41.4% of CRC cases. According to statistics, in 2022, 13,973 patients died from cancer progression (accounting for 5.6% of overall mortality from malignant neoplasms) [2].

The main cause of death in patients with CRC is distant metastases, and despite increasing overall survival rates, metastatic CRC (mCRC) remains a fatal disease, having a 5-year survival rate lower than 15% [3]. More than 80% of CRC metastases are localized in the lymph nodes, liver, and peritoneum [4,5], but CRC metastases can also be found in the brain [6], lungs [7], bones [8], and thyroid gland [9]. Skin involvement occurs in approximately 4% of patients with CRC and is associated with poor survival rates [10]. However, skin metastases of CRC are considered to be a rare event [11] and usually occur during the first 3 years of observation [12]. Most often the metastases are localized in the abdominal and perianal skin [11,13], and there are isolated descriptions of lesions on the face, neck, upper extremities, and penis in the literature [14,15]. Clinical manifestations of cutaneous metastases may include persistent erythema, subcutaneous or intradermal nodules, non-healing ulcers, single or multiple cysts, and granuloma-like or fibroma-like lesions.

Immunohistochemical (IHC) and genetic studies of rectal cancer do not differ from CRC studies and include routine hematoxylin–eosin staining; evaluation of the expression of cytokeratins CK8/18 and CK20, as well as CDX2; and the revelation of activating mutations in proto-oncogenes such as *KRAS*, *NRAS*, and *BRAF*, along with evaluation of the status of microsatellite instability (MSI) [5]. These diagnostic assays are necessary not only to determine the histologic characteristics of the tumor but also to select a treatment regimen, taking into account the possibility of targeted therapy with BRAF inhibitors, checkpoint inhibitors (immunotherapy), etc. [4,5].

The diagnosis of cutaneous metastases is a challenge because colorectal cancer metastases are not the primary clinical hypothesis. This often leads to misdiagnosis, especially in the case of unusual localization (facial or thoracic skin) and in the case of isolated cutaneous lesions. In addition, skin lesions in this group of cancer patients may not only be a manifestation of cutaneous metastasis; rather, they may also be a sign of sarcoidosis or complications of treatment (radiation dermatitis, secondary infections, etc.) [16,17].

Most of the currently available data concerning cutaneous metastases are based on single clinical observations or small case series. Therefore, the accumulation of knowledge about the pathological and molecular features of cutaneous metastases of colorectal cancer is of great clinical importance. Recently, a large series of 29 cutaneous metastases from a wide range of colorectal tumors with particular concerns regarding anatomic localization and the time of onset with respect to primary cancer was reported [18]. Regarding rectal cancer, only 43 cases of cutaneous metastases associated with the primary tumor have been described [11,13]. Very few cases have been characterized at the genetic level.

In this study, we present a detailed description of a particular clinical case of a skin lesion associated with advanced adenocarcinoma of the rectum, including a comprehensive genetic study. We analyze 50 other cases of cutaneous metastases described in the literature, focusing on rectal cancer as an extremely rare phenomenon of metastasis to the skin.

## 2. Materials and Methods

Formaldehyde-fixed paraffin-embedded (FFPE) tissue samples from the same patient were tested. The patient was diagnosed with rectal adenocarcinoma in 2016 and followed until her death in 2022. A tumor sample of the surgical rectal resection, a metastatic tumor from the anal canal, and skin biopsies were collected and evaluated. Hematoxylin and eosin-stained slides were reviewed by two pathologists. Immunohistochemistry (IHC) tests for three markers, namely, Cytokeratin 8/18 (mouse monoclonal antibody, clone L2A1, Cell Marque, Rocklin, CA, USA), Cytokeratin 20 (mouse monoclonal antibody Ks20.8, Cell Marque, Rocklin, CA, USA), and CDX2 (rabbit monoclonal primary antibody EPR2764Y, Cell Marque, Rocklin, CA, USA), were performed.

The levels of carcinoembryonic antigen (CEA) and cancer antigen 19-9 (CA 19-9) were measured using an automated immunoenzymatic analyzer, namely, “Lazurite” (Dynex Technologies, Chantilly, VA, USA). A sample of the patient’s blood was delivered to the laboratory within 15 min after collection in an 8 mL vacuum tube with a silica clotting activator. Centrifugation at 1300 g for 10 min was performed to separate the blood’s formed elements. Levels of CEA > 5 ng/mL and CA 19-9 > 37 U/mL were considered elevated.

Genomic DNA was extracted from FFPE tissues using the QIAamp DNA FFPE Tissue Kit (Qiagen, Hilden, Germany). The microsatellite instability (MSI) assay was performed by PCR amplification of five monomorphic microsatellite loci (NR21, NR24, NR27, BAT25, and BAT26), followed by fragment analysis on the ABI PRISM 3500 genetic analyzer (Thermo Fisher Scientific, Waltham, MA, USA). The obtained data were analyzed using GeneMapper Software 5 (Applied Biosystems, Waltham, MA, USA). If two or more loci were polymorphic, MSI was recorded.

For next-generation sequencing, the genomic DNA was fragmented, and NGS libraries were prepared using the TruSight Cancer Kit (Illumina, San Diego, CA, USA). This panel is a targeted sequencing assay that simultaneously detects and characterizes single-nucleotide variants (SNVs) in 415 genes associated with cancer development. The final library was quantified using the Qubit 4.0 Fluorometer (Thermo Fisher Scientific, Waltham, MA, USA), diluted to a final concentration of 2 nM, and sequenced with the use of NextSeq2000 (Illumina, San Diego, CA, USA). The limit of detection was set to 2–3% variant allele frequency, with mean coverage of ≥200–250 reads.

For Sanger sequencing, the regions of the *BRAF, NRAS, KRAS*, *APC*, *TP53*, and *PTPN11* genes were amplified by standard PCR using the corresponding primer pairs. The purified PCR products were sequenced using the BigDye Terminator v1.1 Cycle Sequencing kit (Thermo Fisher Scientific, Waltham, MA, USA). The samples were analyzed on the ABI PRISM 3500 Genetic Analyzer (Thermo Fisher Scientific, Waltham, MA, USA).

### 2.1. Case Presentation and Clinical Follow-Up

**Figure 1:**
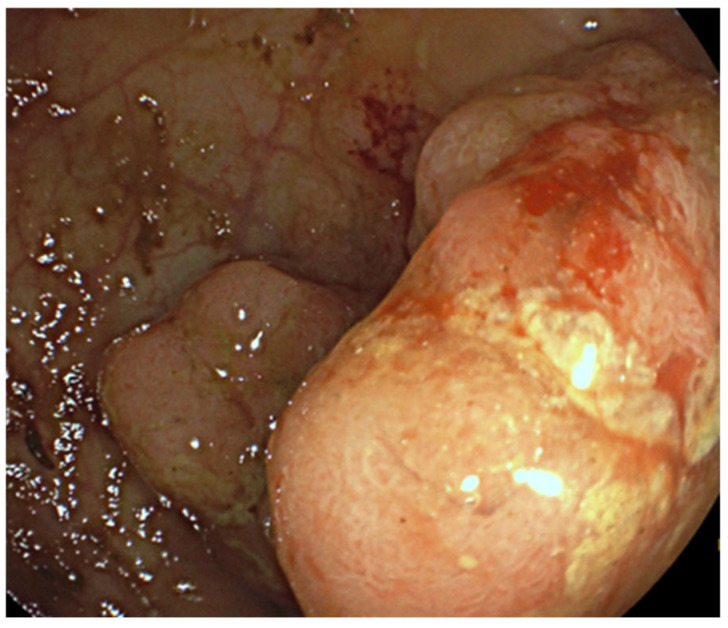
Colonoscopy image obtained during patient examination. An exophytic semi-circular tumor 4.5 × 4 cm in size was visualized in the upper ampullary rectum.

A woman with symptoms and signs of rectal cancer at the age of 56 was diagnosed and treated at the N.N. Blokhin National Medical Research Center of Oncology from 2016 to 2022. Initially, the patient noted the appearance of blood in the stool, pain in the rectum, and constipation. The patient did not have any relatives with cancer in her family. She also did not suffer previously from gastrointestinal diseases. She was a smoker and had been suffering from stage I hypertension since she was 50 years old. She was continuously taking bisoprolol fumarate (5 mg oral tablets) and irbesartan–hydrochlorothiazide in combination (75 mg + 6.25 mg oral tablets) and was adjusted to a blood pressure of 130/85 mmHg. Endoscopic examination revealed an exophytic tumor in the upper ampullary rectum, having a predominantly infiltrative component and a fine bumpy surface structure, which occupied 2/3 of the circumference of the intestine and stenosed the lumen up to 20–22 mm; the tumor size was 4.5 × 4 cm (Figure 1).

A biopsy of the tumor was performed, and the morphological picture corresponded to ulcerated, moderately differentiated adenocarcinoma. Computed tomography (CT) of abdominal cavity organs was performed, and three foci of lesions were visualized in the liver with sizes of 7.5 × 4.5 cm (segment 7 and segment 8), 4.5 × 3.2 cm (segment 8 and segment 5), and 5.0 × 5.0 cm (segment 6).

**Figure 2:**
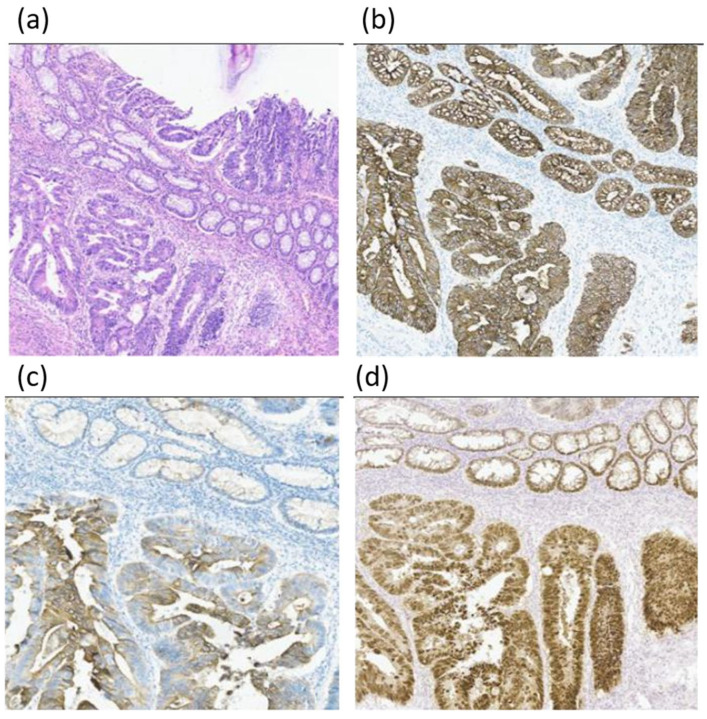
Histological (**a**) and IHC examination (**b**–**d**) of the primary tumor (magnification ×10). Proliferation of glandular and cribriform adenocarcinoma masses with predominance of low-grade structures is detected in the rectum wall. The tumor grows into the myenteron, invading the circular and longitudinal layers. Our IHC study revealed the expression of CK8/18 (**b**), CK20 (**c**), and CDX2 (**d**).

The patient was diagnosed with rectal cancer with liver metastases (T3N0M1a, stage IVa) and underwent 4 cycles of neoadjuvant chemotherapy according to the XELOX scheme (capecitabine and oxaliplatin) with positive dynamics, namely, reduction in liver metastases according to CT scan. In July 2017, right hemihepatectomy, cholecystectomy, and right adrenalectomy were performed. Histological examination of the liver lesions revealed metastases of colon adenocarcinoma, Grade 2 (G2), but there were no tumor elements in the adjacent lymph nodes. After 2.5 months, rectal resection was performed. The morphological pattern was described as adenocarcinoma G2, growing into the myenteron and invading the adjacent fat tissue, with ulceration, and cancer emboli in the vessels were noted (Figure 2a). Immunohistochemistry revealed expression of specific colon cancer markers: CK8/18, CK20, and CDX2 (Figure 2b–d).

Subsequently, 8 cycles of adjuvant chemotherapy (standard XELOX regimen) were carried out. In the next two years of follow-up (from March 2018 to March 2020), there were no signs of relapse or progression. In March 2020, the patient noted pain, burning, and itching in the anus. Excision of the chronic callus of the anterior anal fissure was performed. Histological examination revealed low-grade adenocarcinoma. According to magnetic resonance imaging (MRI), the walls of the anal canal and rectum underwent irregular thickening from 1.1 cm to 5.9 cm; 2.9 × 1.4 cm and 1.3 cm lymph nodes with metastases were found in the inguinal region.

**Figure 3:**
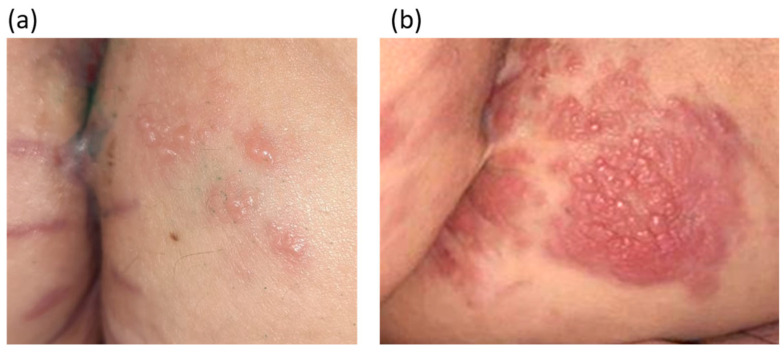
(**a**) First signs of metastatic process on the skin. (**b**) Multiple lenticular and nummular flat dermal papules with a pink color, prone to fusion with the formation of extensive plaques, irregular contours of dense elastic consistency on the skin of the perianal area and the posterior surface of the thighs (October 2020).

The patient underwent complex treatment: chemotherapy with capecitabine alone; radiotherapy on the rectum area, mesorectal tissue, pelvic lymph nodes, and inguinal areas (the total focal dose was 56 Gy); and abdominal–perineal extirpation of the rectum with inguinal lymphadenectomy on both sides. Histological examination revealed adenocarcinoma with germination into the perirectal tissue (without invasion of the sphincter muscles and vaginal wall); adenocarcinoma metastases were detected in two out of the three inguinal lymph nodes. At one month after completion of treatment, a rash appeared on the skin of the perianal area and back of the thighs (Figure 3a). Skin lesions progressed to multiple lenticular and nummular flat dermal papules with a pink color, which were prone to fusion with the formation of extensive plaques but were regarded as anogenital papilloma (Figure 3b). No additional treatments or further examinations were carried out. The patient was placed under dynamic observation.

**Figure 4:**
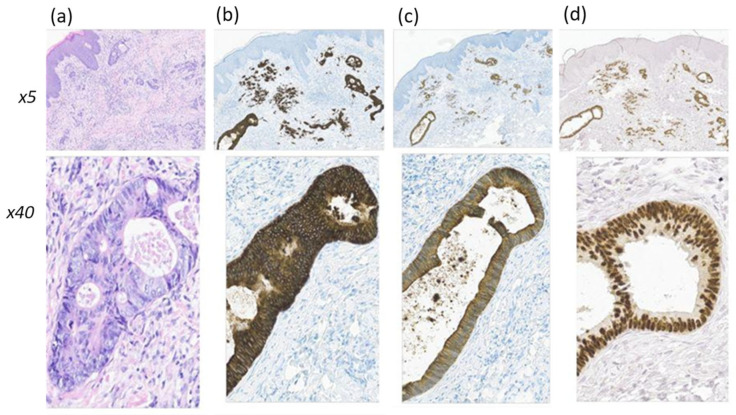
Histological (**a**) and IHC (**b**–**d**) assays of cutaneous metastasis of rectal adenocarcinoma at magnification ×5 and ×40. In the dermis, there are glandular and cribriform adenocarcinoma invasions, represented by structures with low-grade differentiation, as well as solid/trabecular and scirrhous/trabecular structures with cytological atypia, an increase in the number of mitoses, corresponding to intensive proliferation. An IHC study revealed the expression of CK8/18 (**b**), CK20 (**c**), and CDX2 (**d**).

During the next 6 months, the number of elements on the skin increased, so the patient consulted a dermatooncologist. A diagnostic biopsy was performed due to suspected progression of the underlying disease. Histological examination revealed proliferating areas of adenocarcinoma at various degrees of differentiation (Figure 4).

**Figure 5:**
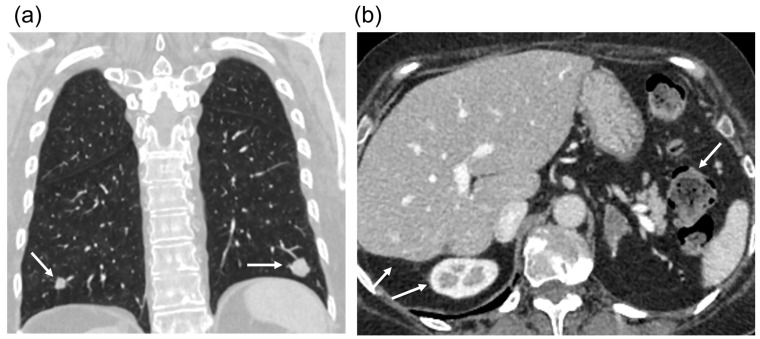
CT scans after 9 cycles of FOLFOX6 chemotherapy. (**a**) CT scan in coronal plane showing lung metastasis (arrows). (**b**) CT scan in venous phase showing the region of liver resection and the absence of the right adrenal gland (two arrows on the left); the left adrenal gland is preserved (arrow on the right).

To treat the metastatic process, 9 cycles of FOLFOX6 chemotherapy (leucovorin, 5-fluorouracil, oxaliplatin) were carried out in combination with bevacizumab. During this course of chemotherapy, CT scan revealed metastases in the lungs (Figure 5).

**Figure 6:**
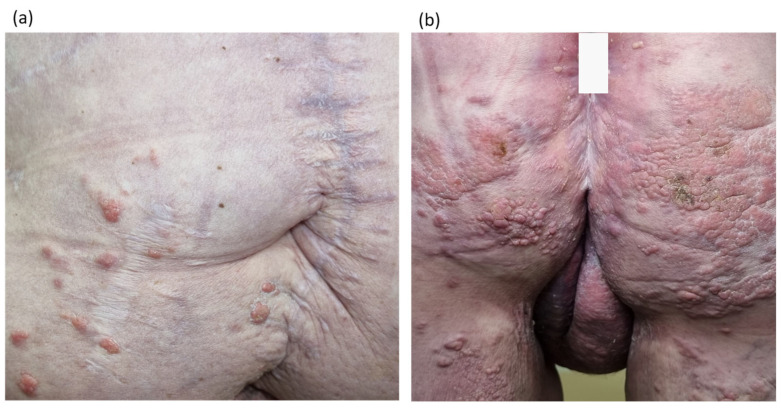
Rectal cancer metastases to the skin. Image showing multiple lenticular pink papules on the abdominal skin (**a**). Image showing the significant increase in the number of lenticular and discoid papules, pinkish cyanotic plaques of irregular shape and dense elastic consistency on the skin of the anogenital area, the back of the thighs (**b**).

Skin symptoms also progressed, with there being a significant increase in the number of pathological elements in the anogenital area, as well as the appearance of multiple pink lenticular papules on the abdominal skin (Figure 6).

**Figure 7:**
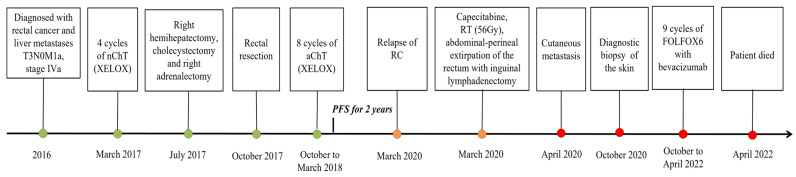
Timeline of the diagnosis and treatment of the patient.

In April 2022, at the age of 62 years, the patient died from the progression of the underlying disease. The results regarding the diagnosis and treatment of the patient are presented in Figure 7.

### 2.2. Laboratory Tests and Molecular Genetic Studies

Laboratory testing of specific oncological markers in peripheral blood was performed at the beginning of the disease and throughout the follow-up period. The level of CEA was high early on in the course of the disease and returned to normal level after treatment of the primary tumor. However, no significant changes in the level of this marker were observed following RC recurrence and the appearance of cutaneous metastases. Values for CA 19-9 corresponded to reference during the entire follow-up period despite disease progression (Table 1).

No mutations in the *BRAF*, *KRAS*, and *NRAS* genes were found by standard genetic testing. Amplification of five microsatellite instability (MSI) markers (NR21, NR24, BAT26, BAT25, and NR27) was performed using genomic DNA extracted from RC sample. The tumor was determined to have microsatellite stability (MSS).

To analyze the mutational profile of the cutaneous metastasis in comparison with primary rectal adenocarcinoma, high-throughput next-generation sequencing (NGS) of the coding regions of 415 cancer-associated genes was performed. The results of the study are provided in Table 2.

**Figure 8:**
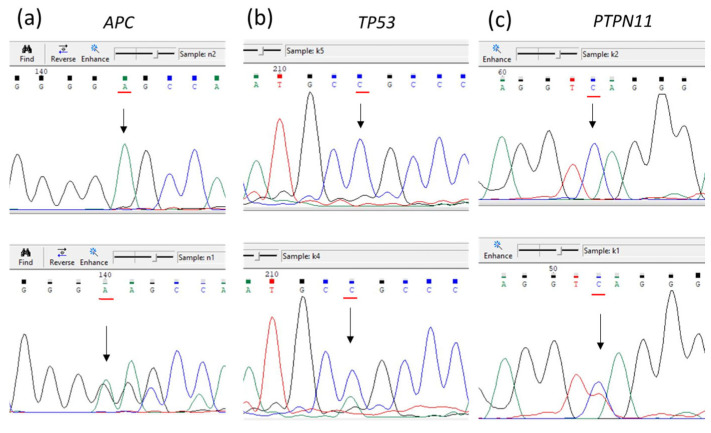
Sanger sequencing verified the presence of somatic mutations in the primary tumor: *APC* gene (**a**), *TP53* gene (**b**), and *PTPN11* gene (**c**). In the upper row, samples from peripheral blood are presented (wildtype); in the lower row, samples from primary adenocarcinoma containing mutations are presented (corresponding nucleotide positions are marked by red lines and arrows).

Pathogenic somatic variants in the *APC*, *TP53*, and *PTPN11* genes were revealed in the primary tumor, but the same genetic variants were absent in cutaneous metastasis. Meanwhile, another pathogenic variant in the *TP53* gene was revealed at a low VAF (11%), and also frameshift variant in the *DYNC1I1* gene encoding cytoplasmic dynein was discovered. The results of NGS findings regarding the primary tumor were confirmed by Sanger sequencing (Figure 8).

## 3. Discussion

We reviewed the literature via open access databases using the following keywords: “cutaneous metastasis”, “skin metastasis”, and “rectal cancer”. A total of 42 full-text articles from 1966 to 2023 describing 50 patients with cutaneous metastasis of rectal cancer were retrieved and analyzed (Table 3).

The sex ratio was 35 males (68.6%) to 16 females (31.4%). The mean age at the time of rectal cancer diagnosis was 57.5 years (19–84 years). The mean time between detection of the primary tumor and cutaneous metastases was 17.9 months; the maximum interval was 14 years. In two patients, secondary skin lesions were diagnosed at the same time as rectal cancer.

Cutaneous metastases were detected in the following areas:

- the inguinal and perianal area, including the penis, scrotum, labia, and vulva, in 29 patients (57%);
- the face, scalp and neck in 13 patients (25%);
- the upper and/or lower extremities in 8 patients (16%);
- the chest and abdominal region, including armpit, in 9 patients (18%);
- the scapular region in 2 patients (4%);
- the back in 2 patients (4%).

In eight cases, there were extensive skin lesions involving several areas.

Metastases to other organs were detected in 29 patients (57%) in the adrenal glands, the lungs, the liver, the brain, the prostate, lymph nodes, and bone and muscular tissues. In 14 cases (27%), no secondary lesions of other organs were detected during the entire follow-up period. The time from detection of cutaneous metastases to patient death was, on average, 8.5 months. At the time of publication, five patients were alive and without signs of progression for seven or more months of follow-up.

It is worth noting that, in eight patients (16%), the histological picture of rectal cancer corresponded to mucinous adenocarcinoma; in three cases (6%), signet ring cell carcinoma was detected, and in one case (2%), small cell carcinoma was detected. For two of the three patients with signet ring cell carcinoma, the diagnosis of RC was made at a young age (29 and 39 years old, respectively), and the secondary skin lesions were disseminated.

Treatment of the patients, in most cases, included rectal resection combined with chemoradiotherapy based on 5-fluorouracil. In more recent studies, the authors used polychemotherapy regimens such as FOLFOX and FOLFIRI, as well as regimens involving monoclonal antibodies (bevacizumab, cetuximab) and targeted therapy (regorafenib, vemurafenib).

In our case, the patient was initially diagnosed with widespread rectal cancer with multiple metastases in the liver, and during additional examination, when skin metastases were detected, associated lung involvement was also revealed. Cutaneous metastases were located in the perianal area and were later detected on the skin of the anterior abdominal wall, which corresponded to the majority of cases. The cutaneous metastases of rectal cancer were not diagnosed in a timely manner, and the patient was observed by a dermatologist for 6 months, receiving symptomatic treatment for papilloma. The patient received a full range of chemotherapy regimens in combination with bevacizumab, and several surgical resections were performed, but the patient died 18 months after the detection of skin metastases.

For patients with CRC, the determination of oncological markers before treatment and at all subsequent stages is needed to assess the efficacy of treatment. A high CEA level before treatment is a negative prognostic factor for both primary tumors and CRC metastases [57]. In further dynamic follow-up, the determination of markers contributes to the early diagnosis of relapse. In our clinical observation, the patient had a highly elevated level of CEA at the beginning of their disease, but upon further laboratory investigations, both markers did not exceed the threshold values either in the case of tumor recurrence or in the case of cutaneous metastases.

Comparison of primary and metastatic tumors in colorectal cancer is used to assess the evolution of a malignant neoplasm (selection of clones, resistance to chemotherapy, radiotherapy, targeted treatment and immunotherapy), the efficacy of treatment, and the approach to personalized treatment. In most cases, the profiles of the primary and metastatic tumors are the same [58,59]; however, for certain markers, there is evidence of a significant discrepancy [60]. In this regard, when a CRC metastasis is detected, it is recommended to carry out an immunohistochemical study, including detection of the mismatch repair deficiency (dMMR) and evaluation of HER2 status, as well as a molecular genetic study, and compare the results with the available data on the primary tumor [60,61].

In IHC studies, 90–95% of tumor samples from patients with CRC have an increased expression of CDX2, which is considered a highly sensitive and specific diagnostic marker for adenocarcinomas of intestinal origin [62]. More than 70% of cutaneous CRC metastases samples show a CK7-negative/CK20-positive molecular profile [63]. We revealed expression of CK8/18, CK20, and CDX2 in both primary and metastatic tumors in our patient, which confirmed the etiology of skin metastasis. Wang et al., in 2019, described MSI in an RC case involving a female with severe subcutaneous back metastasis, indicated by immunohistochemistry markers, such as positivity for MSH2, MSH6, MLH1, and PMS2, which can be used for administering immunotherapy [59].

Activating mutations in the *BRAF* gene are found in 5–9% of all CRC cases [64]. Christensen et al., in 2018, suggested that there is a correlation between the activating mutation V600E in the *BRAF* gene and increased risk of CRC skin metastases [65]. Mutation in the *BRAF* gene may also be associated with loss of CDX2 expression and low cell differentiation [66]. In our case, no mutations in the BRAF gene were detected in the patient’s tumor samples, while CDX2 expression was confirmed, which supports the hypothesis about the correlation between CDX2 expression and the status of the *BRAF* gene.

According to a study by Francesco Sclafani et al., somatic mutations in the *KRAS*, *NRAS*, *BRAF*, *PIK3CA*, and *TP53* genes are encountered in 43%, 9%, 4%, 9%, and 60% of rectal cancer cases, respectively [67]. In our case, a molecular genetic study of the primary tumor material revealed clinically significant somatic variants in the *APC*, *TP53*, and *PTPN11* genes, which is a typical driver event in the initiation of malignant transformation in CRC [67,68].

At the same time, somatic mutations in the *TP53* gene may be associated with extramural vascular invasion, poor regression of the primary tumor, and low 5-year progression-free survival rates [67,69]. The presence of distant metastases in our patient at the time of diagnosis, in combination with a somatic mutation in the *TP53* gene, supports the hypothesis about the effect of these gene mutations on tumor dissemination.

According to clinical studies and literature data, mutations in the *BRAF*, *KRAS*, and *NRAS* genes are associated with an extremely poor prognosis, as well as low efficacy of anti-EGFR therapy, as noted in a comparative study involving patients from a *BRAF/KRAS/NRAS*-wt group [67]. Yazilitas et al., in 2015, described G12D mutation in the *KRAS* gene in an RC involving a female with liver metastasis synchronous to the primary tumor [47]. Despite the absence of mutations in the above genes, the patient described in this article had a relapse of the disease 2 years after the completion of anticancer treatment, and 2 years later, the patient died from progression of the underlying disease.

The combination of MSI and mutation in the *BRAF* gene is more often observed in patients with stage IV colorectal cancer [70], but in such cases, it is possible to use targeted therapy with BRAF inhibitors [71]. The patient under observation in our study was also diagnosed with stage IV rectal cancer, along with low-MSI status (MSS) of the primary tumor and absence of mutations in the *BRAF* gene. MSS-type RC is considered to have limited responsiveness to immune checkpoint inhibitors.

According to Bing Li et al., a mutation in the *APC* gene is associated with a lower tumor mutation burden (TMB) and, as a consequence, with less sensitivity to immunotherapy [68]. The combination of somatic mutations in the *APC* and *TP53* genes in patients with CRC is currently considered as a marker of tumor sensitivity to cetuximab therapy [69]. In our clinical case, at the stage of obtaining the results of the molecular genetic study, the patient was already receiving palliative therapy, and the use of the options described above was impossible.

In the patient’s cutaneous metastasis sample, based on our observations, a different pathogenic mutation in the *TP53* gene was found compared to the primary tumor. Despite the difference in mutation profiles between the primary tumor and the skin metastasis, the histological and IHC pictures were identical. The results obtained may be due to the elimination, during intensive polychemotherapy, of the main clone of cells carrying mutations inherent in the primary tumor. Additionally, a variant of unknown clinical significance in the *DYNC1I1* gene was identified in the patient’s metastatic tumor, resulting in a frameshift, and may have influenced protein function. Today, the role of the *DYNC1I1* gene in carcinogenesis is not fully known. The results of some studies show a potential prognostic role for this biomarker in tumors from various localizations [72,73].

Thus, significant molecular differences were revealed between the primary and metastatic tumor samples in the presented clinical case. A similar result was described in tumor genome study [74], as well as in a study of the CRC transcriptome [75]. The identified differences may be associated with both the heterogeneity of the malignant neoplasm and the evolution of the primary tumor on top of previous treatment. In this regard, the study of all metastatic lesions, as well as assaying of circulating tumor DNA, will provide more complete and unbiased information about the molecular profile of the metastatic tumor.

## 4. Conclusions

Metastases of rectal cancer to the skin are extremely rare; they can be either the earliest or latest manifestation of the disease and can masquerade as benign skin neoplasms. The differential diagnosis should be made with sarcoidosis or complications of treatment (radiation dermatitis and secondary infections). Cutaneous metastases of rectal cancer are most often found in the inguinal and perianal regions, as well as on the face, scalp, neck, and other parts of the body. In more than half of cases, cutaneous metastases are combined with metastases to other organs. The development of cutaneous metastases is associated with a poor prognosis, and the average time from detection of skin lesions to patient death is about 8.5 months. In this regard, a thorough examination of the skin of patients with rectal cancer and the identification of cutaneous metastases are necessary steps in monitoring patients who have undergone treatment for the primary tumor. At the same time, the possibility of clonal evolution of the primary tumor and alteration of the mutational profile during disease progression indicates the need for additional molecular genetic studies on cutaneous metastases to select the optimal therapeutic approach.

## Figures and Tables

**Table 1:** Level of oncological markers in association with the stage of disease (CEA—carcinoembryonic antigen; CA 19-9—cancer antigen 19.9).

Marker/Data	Reference Value	26 December 2016 (Before Treatment)	19 May 2017 (After nChT)	26 September 2018 (Follow-Up with No Relapse)	31 March 2020 (Relapse of RC)	19 August 2020 (Cutaneous Metastases)
CEA (ng/mL)	≤5,093	35	10.21	5.52	4.32	4.96
CA19-9 (U/mL)	0–37	–	–	–	9.54	12.3

**Table 2:** Comparison of the mutational profiles in DNA samples isolated from lymphocytes of peripheral venous blood, primary tumors, and cutaneous metastasis.

Sample	Gene	Nucleotide Variant (Protein Variant)	Pathogenicity (ACMG)	VAF (%)
Blood	No clinically significant variants		N/A	N/A
Primary tumor (adenocarcinoma)	*APC*	c.3317del (p.Gly1106Glufs)	Pathogenic	50%
	*TP53*	c.733G > T (p.Gly245Cys)	Pathogenic	58%
	*PTPN11*	c.1505C > T (p.Ser502Leu)	Pathogenic	51%
Cutaneous metastasis	*TP53*	c.524G > A (p.Arg175His)	Pathogenic	11%
	*DYNC1I1*	c.1748del (p.Asn583Ilefs)	Uncertain	10%

abbreviations: ACMG—the American College of Medical Genetics and Genomics; *APC*—adenomatous polyposis coli gene; *DYNC1I1*—dynein cytoplasmic 1 intermediate chain 1 gene; N/A—not available; *TP53*—tumor protein 53 gene; *PTPN11*—protein tyrosine phosphatase non-receptor type 11 gene; VAF—variant allele frequency.

**Table 3:** Clinical cases of cutaneous metastasis (Cmts) of rectal cancer (RC), including information such as age at diagnosis of RC (AC—adenocarcinoma, MAC—mucinous adenocarcinoma, mts—metastasis, aRT—adjuvant radiation therapy, aChRT—adjuvant chemo- and radiation therapy, aChT—adjuvant chemotherapy, RT—radiation therapy, nRT—neoadjuvant radiation therapy, nChT—neoadjuvant chemotherapy, ChRT—chemo- and radiation therapy, ST—surgical treatment, ChT—chemotherapy, 5-FU—5-fluorouracil, PT—palliative treatment, PRT—palliative radiation therapy, N/A—not available, *—treatment of RC, and **—treatment of Cmts).

Author, Year	Sex	Age, Year	Interval (RC-Cmts)	Cmts Site	Treatment * RC, ** Cmts	Mts to Other Organs	Time from Cmts	Comment
Reingold et al., 1966 [19]	m	N/A	N/A	Big toe, chest, and abdomen	N/A	N/A	3 m	no
De Friend et al., 1992 [20]	f	49	7 m	Perianal area	* ST	N/A	N/A	no
Sukumar et al., 2001 [21]	m	75	3 m	Scrotum and penis	* ST, 6 courses ChT (5-FU), RT	Absent	2 m	no
Melis et al., 2002 [22]	m	41	1 m	Anterior pelvic region and perineum	nChT	Prostate, liver	N/A	no
Damin et al., 2003 [23]	m	44	6 m	Suprapubic region and groin	* ST ** local RT	Lung, 2 m after Cmts	5 m	Similar to herpes zoster
Hayashi et al., 2003 [24]	m	50	4 m	Scrotum	ST	Absent	7 m	Signet ring cell cancer
Sarid et al., 2004 [25]	f	60	16 m	Abdomen and armpit on the left	* nRT, ST, RT, 12 cycles of ChT (5-FU) ** ST	Absent	56 m	MAC
Torné et al., 2006 [26]	m	62	0 m	Iliac, pubic, inguinal areas, left thigh	RT, ChT	N/A	N/A	Similar to herpes zoster
Tan et al., 2006 [27]	m	70	22 m	Left scapula	* ST, aChRT ** PT	Absent	N/A	MAC
	m	51	10 m	Right scapula	* ST, aChT ** PT	Lymph nodes and lung, 14 m after RC	7 m	MAC
	f	53	20 m	Left labia	* ST, aChRT ** ChTT	Lung and brain	26 m	no
Kilickap et al., 2006 [28]	m	29	14 m	Chest, left armpit	*ST, aChRT ** ChT (5-FU)	Liver, 4 m after Cmts	N/A	Signet ring cell cancer
Nasty et al., 2007 [29]	f	76	4 m	Parotid skin and frontal face	nChRT	Lung after 10 m	12 m	no
Tranchart et al., 2008 [30]	f	59	14 m	Perianal area	* ST ** ST	Para-aortic lymph nodes 16 m after Cmts	N/A	no
Tranchart et al., 2008 [30]	m	70	10 m	Perianal area	* nChRT, ST * ST	Absent	N/A	no
García Muñoz et al., 2008 [31]	f	57	20 m	Scalp	* ST ** ChT (5-FU), RT	Lung synchronous to Cmts	Alive after 10 m of follow-up	no
Vijayasekar et al., 2008 [32]	f	47	168 m	Colostomy area	* ST, aRT ** ST	Absent	N/A	no
Gazoni et al., 2008 [33]	m	55	0 m	Perineum and scrotum	ST, aChRT, RT	Liver and lung	3 m	no
	f	66	0 m	Labial fold and perineum	ST, aChRT, RT	Lung	4 m	no
	m	68	0 m	Inner thigh and arm	ST, aChRT, RT	Retro-peritoneum	3 m	no
	m	72	0 m	Scrotum	ST, aChRT, RT	Absent	5 m	no
	m	65	0 m	Penis	ST, aChRT, RT	Absent	7 m	no
	m	78	0 m	Scrotum	ST, aChRT, RT	Liver	1 m	no
Ayadi et al., 2009 [34]	m	63	5 m	Scalp	* Refuse **4 cycles ChT (etoposide, cisplatin)	Liver synchronous to Cmts	9 m	Small cell cancer
Vilbergsson et al., 2010 [35]	m	82	2 m	Left cheek	* ST	Absent	N/A	no
Saladzinskas et al., 2010 [36]	m	65	42 m	Upper lip	* nRT, ST, aChT(5-FU) ** ST	Lung synchronous to Cmts	Alive after 7 m of follow-up	MAC
Goris et al., 2011 [37]	m	79	33 m	Pubis, pelvis, scrotum	* ST	Presacral area 24 m after ST	6 m	no
Balta et al., 2012 [38]	m	45	12 m	Left inguinal and perianal areas	* ST	Absent	N/A	MAC
Harp et al., 2012 [39]	f	48	N/A	Chin	N/A	Lung, brain, vertebrae	N/A	no
Balta et al., 2013 [40]	f	84	0 m	Occipital area	* nChRT	N/A	0 m	no
Hashimi et al., 2013 [41]	m	70	48 m	Left cheek	* nChT, ST ** ST	Lung, 24 m after RC	Alive at publication date	no
de Miguel Valencia et al., 2013 [42]	m	55	18 m	Pubic area, left armpit, lower extremities	* nChRT, ST, aChT (capecitabine)	Lung and liver synchronous to Cmts	0 m	MAC
Ozgen et al., 2013 [43]	m	65	22 m	Scrotum	* nChRT, ST, aChRT ** ChT (capecitabine), RT	Absent	Alive after 12 m of follow-up	no
Akpak et al., 2014 [44]	f	44	36 m	Vulvar area	* ST ** ST	N/A	N/A	no
Win et al., 2015 [45]	m	68	0 m	Forehead	N/A	Lung, liver, bone, gluteal muscle synchronous to Cmts	N/A	no
Fabiani, 2015 [46]	m	73	60 m	Penis	* ST, aRT ** ST, ChT	Absent	19 m	Cmts with RC relapse
Yazilitas et al., 2015 [47]	f	50	18 m	Forehead	* nChT, ST, aChRT	Liver	N/A	KRAS G12D
Liasis et al., 2016 [48]	m	61	2 m after completing nChRT	Perineum	* nChRT ** ST, aChT	Absent	Alive after 60 m	no
Wu et al., 2016 [49]	m	34	28 m	Scrotum	* ST ** ChT (gemcitabine, docetaxel)	Liver, 12 m after Cmts	12 m	no
Dehal et al., 2016 [11]	m	47	12 m	Groin and perineum	* ChRT ** PT	Lymph nodes	Alive after 12 m	MAC
Wang et al., 2017 [50]	f	76	1 m	Back	* ST	N/A	2 m	MSH2(+) MSH6(+)MLH1(+)PMS2(+)
Hamid et al., 2018 [51]	f	75	14 m	Perineum	* nChT, ST	N/A	N/A	no
Yagnik et al., 2018 [15]	m	38	18 m	Pubic region, penis	* ST, ChT (FOLFOX) ** Refuse	Liver	2 m	no
Malla et al., 2019 [52]	m	35	2 m	Face, chest, abdomen, back	* ST, aChT ** ST	Liver synchronous to Cmts	3 m	MAC
Morales-Cruz et al., 2019 [53]	m	39	3 m	Skin flap, face, abdomen and lower extremities	* nChT (5-FU), RT, ChT (FOLFOX4), ST ** ST, ChT (FOLFIRI, FEC-7)	Vertebrae, peritoneum	N/A	Signet ring cell cancer
Hakami et al., 2020 [13]	m	45	0 m	Inguinal area and peritenium	* PRT, ST	Lung, lymph nodes synchronous to Cmts	Death due to pneumonia	no
Samanci et al., 2020 [14]	m	45	N/A	Scalp and lower jaw	* nChT (5-FU, oxaliplatin, cetuximab, irinotecan, bevacizumab, regorafenib) ** PT	Adrenal glands, lung, left parietal bone	3 m	no
Zhou et al., 2021 [54]	f	53	8 m	Abdomen and perineum	* nChT (FOLFOX, cetuximab) ** ChT (FOLFIRI, bevacizumab, cetuximab, vemurafenib)	Liver, vertebrae, gluteal muscle, lymph nodes synchronous to Cmts	5 m	*BRAF* V600E
Alina et al., 2023 [55]	m	65	108 m	Lower and upper extremities	* ST, aChRT ** PT	N/A	4 m	Synchronous prostate cancer
Akhtar et al., 2024 [56]	m	19	7 m	Neck	* nChRT	Absent	Alive at the time of publication	no
Present study, 2024	f	56	42 m	Perianal area, thighs, abdomen	* nChT (XELOX), ST ** ChT (FOLFOX6), bevacizumab	Liver, lymph nodes before Cmts, lung after Cmts	24 m	AC G2

## Data Availability

The original contributions of the study are included in the article. Further inquiries can be directed to the corresponding author.

## References

[1] Ferlay J., Ervik M., Lam F., Laversanne M., Colombet M., Mery L., Piñeros M., Znaor A., Soerjomataram I., Bray F. (2024). Global Cancer Observatory: Cancer Today.

[2] Kaprin A.D., Starinsky V.V., Petrova G.V. (2023). Malignant Neoplasms in Russia in 2022 (Morbidity and Mortality).

[3] Rumpold H., Niedersüß-Beke D., Heiler C., Falch D., Wundsam H.V., Metz-Gercek S., Piringer G., Thaler J. (2020). Prediction of mortality in metastatic colorectal cancer in a real-life population: A multicenter explorative analysis. BMC Cancer.

[4] Biller L.H., Schrag D. (2021). Diagnosis and Treatment of Metastatic Colorectal Cancer: A Review. JAMA.

[5] Shin A.E., Giancotti F.G., Rustgi A.K. (2023). Metastatic colorectal cancer: Mechanisms and emerging therapeutics. Trends Pharmacol. Sci..

[6] Michl M., Thurmaier J., Schubert-Fritschle G., Wiedemann M., Laubender R.P., Nüssler N.C., Ruppert R., Kleeff J., Schepp W., Reuter C. (2015). Brain Metastasis in Colorectal Cancer Patients: Survival and Analysis of Prognostic Factors. Clin. Colorectal Cancer.

[7] Dai W., Guo C., Wang Y., Li Y., Xie R., Wu J., Yao B., Xie D., He L., Li Y. (2023). Identification of hub genes and pathways in lung metastatic colorectal cancer. BMC Cancer.

[8] Fornetti J., Welm A.L., Stewart S.A. (2018). Understanding the Bone in Cancer Metastasis. J. Bone Miner. Res..

[9] Ciriano Hernández P., Martínez Pinedo C., Calcerrada Alises E., García Santos E., Sánchez García S., Picón Rodríguez R., Jiménez Higuera E., Sánchez Peláez D., Herrera Montoro V., Martín Fernández J. (2020). Colorectal cancer metastases to the thyroid gland: A case report. World J. Gastrointest. Surg..

[10] Bittencourt M.J.S., Imbiriba A.A., Oliveira O.A., Santos J.E.B.D. (2018). Cutaneous metastasis of colorectal cancer. An. Bras. Dermatol..

[11] Dehal A., Patel S., Kim S., Shapera E., Hussain F. (2016). Cutaneous Metastasis of Rectal Cancer: A Case Report and Literature Review. Perm. J..

[12] Gmitter T.L., Dhawan S.S., Phillips M.G., Wiszniak J. (1990). Cutaneous metastases of colonic adenocarcinoma. Cutis.

[13] Hakami R., Alali M.N., Alshammari T., AlShammari S., Alyahya Z., Ayesh M., AlSaad K., Abduljabbar A. (2020). A cutaneous metastasis of unresectable rectal adenocarcinoma: A case report and literature review. Int. J. Surg. Case Rep..

[14] Samanci N.S., Akdogan S., Celik E., Kutlu O., Ulgen O.A., Demirelli F.H. (2020). Facial cutaneous metastasis of rectal adenocarcinoma. North. Clin. Istanb..

[15] Yagnik V.D. (2018). Penile and multiple cutaneous metastases over the pubic region from a rectal adenocarcinoma: An uncommon case. Ci Ji Yi Xue Za Zhi.

[16] Liu S., Wang Y.L., Shi S.T., Zeng G.D., Song Y.W., Zhang X.D., Zheng J., Fan X.J., Liu Y.P. (2022). The effect of recombinant human epidermal growth factor on radiation dermatitis in rectal and anal cancer patients: A self-controlled study. BMC Cancer.

[17] Dundar A., Dundar B., Inanc M., Canoz O., Oymak F.S., Abdulrezzak U. (2019). Sarcoidosis with Multiorgan Involvement and Cutaneous Manifestations after Colonic Adenocarcinoma Resection. Indian J. Nucl. Med..

[18] Parente P., Ciardiello D., Reggiani Bonetti L., Famiglietti V., Cazzato G., Caramaschi S., Attino V., Urbano D., Di Maggio G., Ingravallo G. (2021). Cutaneous Metastasis from Colorectal Cancer: Making Light on an Unusual and Misdiagnosed Event. Life.

[19] Reingold I.M. (1966). Cutaneous metastases from internal carcinoma. Cancer.

[20] De Friend D.J., Kramer E., Prescott R., Corson J., Gallagher P. (1992). Cutaneous perianal recurrence of cancer after anterior resection using the EEA stapling device. Ann. R. Coll. Surg. Engl..

[21] Sukumar N., Qureshi A. (2001). Adenocarcinoma of rectum metastasizing to penis. Med. J. Malays..

[22] Melis M., Scintu F., Marongiu L., Mascia R., Frau G., Casula G. (2002). Inflammatory Cutaneous Metastasis from Rectal Adenocarcinoma. Dis. Colon Rectum.

[23] Damin D.L., Lazzaron A.R., Tarta C., Cartel A., Rosito M.A. (2003). Massive zosteriform cutaneous metastasis from rectal carcinoma. Tech. Coloproctol..

[24] Hayashi H., Shimizu T., Shimizu H. (2003). Scrotal metastases originating from colorectal carcinoma. Clin. Exp. Dermatol..

[25] Sarid D., Wigler N., Gutkin Z., Merimsky O., Leider-Trejo L., Ron I.G. (2004). Cutaneous and subcutaneous metastases of rectal cancer. Int. J. Clin. Oncol..

[26] Torné J., Bonaut B., Sanz C., Martínez C., Torrero M.V., Miranda-Romero A. (2006). Metástasis cutáneas de adenocarcinoma de recto con distribución herpetiforme [Cutaneous metastases of rectal adenocarcinoma in a herpetiform distribution]. Actas Dermosifiliogr..

[27] Tan K.Y., Ho K.S., Lai J.H., Lim J.F., Ooi B.S., Tang C.L., Eu K.W. (2006). Cutaneous and subcutaneous metastases of adenocarcinoma of the colon and rectum. Ann. Acad. Med. Singap..

[28] Kilickap S., Aksoy S., Dinçer M., Saglam E.A., Yalçin S. (2006). Cutaneous metastases of signet cell carcinoma of the rectum without accompanying visceral involvement. South. Med. J..

[29] Nasti G., Facchini G., Caraglia M., Franco R., Mura A.L., Staiano M., Budillon A., Iaffaioli R.V., Ottaiano A. (2007). Concomitant Occurrence of Facial Cutaneous and Parotid Gland Metastases from Rectal Cancer after Preoperative Chemoradiotherapy. Oncol. Res. Treat..

[30] Tranchart H., Benoist S., Penna C., Julie C., Rougier P., Nordlinger B. (2008). Cutaneous Perianal Recurrence on the Site of Lone Star Retractor™ after J-pouch Coloanal Anastomosis for Rectal Cancer: Report of Two Cases. Dis. Colon Rectum.

[31] García Muñoz E., Oyarzo M., Pinedo G. (2008). Metástasis cutáneas de cáncer rectal [Cutaneous metastasis of rectal cancer]. Cir. Esp..

[32] Vijayasekar C., Noormohamed S., Cheetham M.J. (2008). Late recurrence of large peri-stomal metastasis following abdomino-perineal resection of rectal cancer. World J. Surg. Oncol..

[33] Gazoni L.M., Hedrick T.L., Smith P.W., Friel C.M., Swenson B.R., Adams J.D., Lisle T.C., Foley E.F., Ledesma E.J. (2008). Cutaneous Metastases in Patients with Rectal Cancer: A Report of Six Cases. Am. Surg..

[34] Ayadi L., Zribi J., Mziou T.J., Ellouz S., Khabir A., Bahri I., Turki H., Sellami-Boudawara T. (2009). Métastase au niveau du cuir chevelu d’un carcinome a petites cellules du rectum: Un cas inhabituel [Scalp metastasis from small cell carcinoma of the rectum: An unusual case]. Tunis. Med..

[35] Vilbergsson E., Isaksson H.J., Möller P.H. (2010). Sjúkratilfelli: Meinvarp frá endaþarms-krabbameini í andliti [Case report: Facial skin metastasis from rectal adenocarcinoma]. Laeknabladid.

[36] Saladzinskas Z., Tamelis A., Paskauskas S., Pranys D., Pavalkis D. (2010). Facial skin metastasis of colorectal cancer: A case report. Cases J..

[37] Goris Gbenou M.C., Wahidy T., Llinares K., Cracco D., Perrot A., Riquet D. (2011). Atypical Phimosis Secondary to a Preputial Metastasis from Rectal Carcinoma. Case Rep. Oncol..

[38] Balta I., Vahaboglu G., Karabulut A.A., Yetisir F., Astarci M., Gungor E., Eksioglu M. (2012). Cutaneous metastases of rectal mucinous adenocarcinoma mimicking granuloma inguinale. Intern. Med..

[39] Harp J.L., Marcus R., Husain S., Grossman M.E. (2012). Metastatic cystic nodule of rectal SCC with basaloid features mimicking a BCC of the face. J. Am. Acad. Dermatol..

[40] Balta A.Z., Sücüllü I., Özdemir Y., Dandin Ö. (2013). A rare clinical manifestation ofrectal adenocarcinoma and synchronous scalp metastasis: A case report. Turk. J. Surg..

[41] Hashimi Y., Dholakia S. (2013). Facial cutaneous metastasis of colorectal adenocarcinoma. BMJ Case Rep..

[42] de Miguel Valencia M.J., Fraile González M., Yagüe Hernando A., Oteiza Martínez F., Ciga Lozano M.A., Armendáriz Rubio P., de Miguel Velasco M., Ortiz Hurtado H. (2013). Metástasis cutáneas de cáncer de recto [Cutaneous metastases of rectal cancer]. An. Sist. Sanit. Navar..

[43] Ozgen A., Karakaya E., Bozdoğan N. (2013). Scrotal skin metastasis from rectum adenocarcinoma. Rare Tumors.

[44] Akpak Y.K., Dandin Ö., Gün İ., Atay V., Haholu A. (2014). A rare case of vulvar skin metastasis of rectal cancer after surgery. Int. J. Dermatol..

[45] Win A.Z., Aparici C.M. (2015). A case of metastatic rectal squamous cell carcinoma initially diagnosed as lung cancer. J. Clin. Imaging Sci..

[46] Fabiani A., Filosa A., Fioretti F., Mammana G. (2015). Penile plaque as predictor of an advanced anorectal carcinoma: A case report. Arch. Ital. Urol. Androl..

[47] Yazilitas D. (2015). Rectal cancer with metastasis to the face. Int. J. Hematol. Oncol..

[48] Liasis L., Papaconstantinou H.T. (2016). Colorectal cancer implant in an external hemorrhoidal skin tag. Bayl. Univ. Med. Cent. Proc..

[49] Wu G., Gu B.J., Nastiuk K.L., Gu J., Wu D.L. (2016). Metastasis to scrotal skin as the initial manifestation in a patient with rectal adenocarcinoma: A rare case report and literature review. Asian J. Androl..

[50] Wang D.Y., Ye F., Lin J.J., Xu X. (2017). Cutaneous metastasis: A rare phenomenon of colorectal cancer. Ann. Surg. Treat. Res..

[51] Hamid M., Majbar A.M., Hrora A., Ahallat M. (2017). Perineal skin recurrence on the site of Lone Star Retractor: Case report. Surg. Case Rep..

[52] Malla S., Razik A., Vyas S. (2019). Cutaneous metastasis in adenocarcinoma rectum. BMJ Case Rep..

[53] Morales-Cruz M., Salgado-Nesme N., Trolle-Silva A.M., Rodríguez-Quintero J.H. (2019). Signet ring cell carcinoma of the rectum: Atypical metastatic presentation. BMJ Case Rep..

[54] Zhou S., Tang W., Wang Q., Zhang X., Jin X., Xu X., Fu J. (2021). A Case Report: Cutaneous Metastasis of Advanced Rectal Cancer with BRAF Mutation. Onco Targets Ther..

[55] Alina V., Maghiar O., Maghiar L., Cuc R., Pop O., Pascalau A., Boros M., Maghiar A. (2023). Cutaneous metastasis of rectal adenocarcinoma: A case report and literature review. Pol. J. Pathol..

[56] Akhtar S., Khan Q., Rehman A., Khalid M.W., Siddique K. (2024). A Rare Case of Cutaneous Metastasis of Unresectable Rectal Adenocarcinoma. Cureus.

[57] Allard M.A., Adam R., Giuliante F., Lapointe R., Hubert C., Ijzermans J.N.M., Mirza D.F., Elias D., Laurent C., Gruenberger T. (2017). Long-term outcomes of patients with 10 or more colorectal liver metastases. Br. J. Cancer.

[58] Brannon A.R., Vakiani E., Sylvester B.E., Scott S.N., McDermott G., Shah R.H., Kania K., Viale A., Oschwald D.M., Vacic V. (2014). Comparative sequencing analysis reveals high genomic concordance between matched primary and metastatic colorectal cancer lesions. Genome Biol..

[59] Wang Z., Tang X., Wu X., Yang M., Wang D. (2019). Mismatch repair status between primary colorectal tumor and metastatic tumor, a retrospective consistent study. Biosci. Rep..

[60] Shan L., Lv Y., Bai B., Huang X., Zhu H. (2018). Variability in HER2 expression between primary colorectal cancer and corresponding metastases. J. Cancer Res. Clin. Oncol..

[61] Haraldsdottir S., Roth R., Pearlman R., Hampel H., Arnold C.A., Frankel W.L. (2016). Mismatch repair deficiency concordance between primary colorectal cancer and corresponding metastasis. Fam. Cancer.

[62] Saad R.S., Ghorab Z., Khalifa M.A., Xu M. (2011). CDX2 as a marker for intestinal differentiation: Its utility and limitations. World J. Gastrointest. Surg..

[63] Saeed S., Keehn C.A., Morgan M.B. (2004). Cutaneous metastasis: A clinical, pathological, and immunohistochemical appraisal. J. Cutan. Pathol..

[64] Van Cutsem E., Köhne C.H., Láng I., Folprecht G., Nowacki M.P., Cascinu S., Shchepotin I., Maurel J., Cunningham D., Tejpar S. (2011). Cetuximab plus irinotecan, fluorouracil, and leucovorin as first-line treatment for metastatic colorectal cancer: Updated analysis of overall survival according to tumor KRAS and BRAF mutation status. J. Clin. Oncol..

[65] Christensen T.D., Palshof J.A., Larsen F.O., Poulsen T.S., Høgdall E., Pfeiffer P., Jensen B.V., Yilmaz M.K., Nielsen D. (2018). Associations between primary tumor RAS, BRAF and PIK3CA mutation status and metastatic site in patients with chemoresistant metastatic colorectal cancer. Acta Oncol..

[66] Aasebø K., Dragomir A., Sundström M., Mezheyeuski A., Edqvist P.H., Eide G.E., Ponten F., Pfeiffer P., Glimelius B., Sorbye H. (2020). CDX2: A prognostic marker in metastatic colorectal cancer defining a better BRAF mutated and a worse KRAS mutated subgroup. Front. Oncol..

[67] Sclafani F., Wilson S.H., Cunningham D., Gonzalez De Castro D., Kalaitzaki E., Begum R., Wotherspoon A., Capdevila J., Glimelius B., Roselló S. (2020). Analysis of KRAS, NRAS, BRAF, PIK3CA and TP53 mutations in a large prospective series of locally advanced rectal cancer patients. Int. J. Cancer.

[68] Li B., Zhang G., Xu X. (2023). APC mutation correlated with poor response of immunotherapy in colon cancer. BMC Gastroenterol..

[69] Thota R., Yang M., Pflieger L., Schell M.J., Rajan M., Davis T.B., Wang H., Presson A., Pledger W.J., Yeatman T.J. (2021). APC and TP53 Mutations Predict Cetuximab Sensitivity across Consensus Molecular Subtypes. Cancers.

[70] Li Z.N., Zhao L., Yu L.F., Wei M.J. (2020). BRAF and KRAS mutations in metastatic colorectal cancer: Future perspectives for personalized therapy. Gastroenterol. Rep..

[71] Ducreux M., Chamseddine A., Laurent-Puig P., Smolenschi C., Hollebecque A., Dartigues P., Samallin E., Boige V., Malka D., Gelli M. (2019). Molecular targeted therapy of BRAF-mutant colorectal cancer. Ther. Adv. Med. Oncol..

[72] Zhou J., Zhu Y., Ma S., Li Y., Liu K., Xu S., Li X., Li L., Hu J., Liu Y. (2021). Bioinformatics analysis identifies DYNC1I1 as prognosis marker in male patients with liver hepatocellular carcinoma. PLoS ONE.

[73] Ananya R.G., Pandi C., Kannan B., Pandi A., Prasad P., Jayaseelan V.P., Arumugam P. (2023). DYNC1I1 acts as a promising prognostic biomarker and is correlated with immune infiltration in head and neck squamous cell carcinoma. J. Stomatol. Oral. Maxillofac. Surg..

[74] Puccini A., Xiu J., Goldberg R.M., Grothey A., Shields A.F., Salem M.E., Seeber A., Battaglin F., Berger M.D., El-Deiry W.S. (2019). Molecular differences between lymph nodes (LNs) and distant metastases (mets) in colorectal cancer (CRC). J. Clin. Oncol..

[75] Kamal Y., Schmit S.L., Hoehn H.J., Amos C.I., Frost H.R. (2019). Transcriptomic differences between primary colorectal adenocarcinomas and distant metastases reveal metastatic colorectal cancer subtypes. Cancer Res..

